# Increased postexercise insulin sensitivity is accompanied by increased AS160 phosphorylation in slow‐twitch soleus muscle

**DOI:** 10.14814/phy2.12162

**Published:** 2014-12-11

**Authors:** Maiko Iwabe, Emi Kawamoto, Keiichi Koshinaka, Kentaro Kawanaka

**Affiliations:** Department of Health and Nutrition, Niigata University of Health and Welfare, Niigata, Japan

**Keywords:** AS160, exercise, glucose uptake, insulin sensitivity, slow‐twitch muscle, TBC1D1

## Abstract

A single bout of exercise can enhance insulin‐stimulated glucose uptake in both fast‐twitch (type II) and slow‐twitch (type I) skeletal muscle for several hours postexercise. Akt substrate of 160 kDa (AS160) is most distal insulin signaling proteins that have been proposed to contribute to the postexercise enhancement of insulin action in fast‐twitch muscle. In this study, we examined whether the postexercise increase in insulin action of glucose uptake in slow‐twitch muscle is accompanied by increased phosphorylation of AS160 and its paralog TBC1D1. Male Wistar rats (~1‐month‐old) were exercised on a treadmill for 180 min (9 m/min). Insulin (50 μU/mL)‐stimulated glucose uptake was increased at 2 h after cessation of exercise in soleus muscle composed of predominantly slow‐twitch fibers. This postexercise increase in insulin action of glucose uptake was accompanied by increased phosphorylation of AS160 (detected by phospho‐Thr642 and phospho‐Ser588 antibody). On the other hand, prior exercise did not increase phosphorylation of TBC1D1 (detected by phospho‐Thr590) at 2 h postexercise. These results suggest the possibility that an enhancement in AS160 phosphorylation but not TBC1D1 phosphorylation is involved with increased postexercise insulin action of glucose uptake in slow‐twitch muscle.

## Abstract (precis)

In slow‐twitch soleus muscle, phosphorylation of AS160 Thr642 and Ser588 was increased together with the enhanced insulin action of the glucose uptake at 2 h postexercise. The phosphosite of TBC1D1 (Thr590), which is possibly important for insulin‐stimulated glucose uptake, did not increase phosphorylation at 2 h postexercise. These results suggest that the increased phosphorylation of AS160, but not TBC1D1, can account for the postexercise enhancement in the insulin action of the glucose uptake in slow‐twitch muscle.

## Introduction

Skeletal muscle is the largest tissue in the human body by mass and is the major site of insulin‐stimulated glucose disposal (DeFronzo et al. 1981). In skeletal muscle, insulin stimulation causes the translocation of GLUT4 glucose transporters from intracellular regions to the plasma membrane and the t‐tubule system, leading to the facilitation of glucose uptake in skeletal muscle. Insulin's proximal signaling events include activation of the insulin receptor, insulin receptor substrates, phosphatidylinositol (PI) 3‐kinase, and Akt. In rodent skeletal muscle, Akt phosphorylates the Akt substrate of 160 kDa (AS160; also known as TBC1D4) (Bruss et al. 2005) and its paralog TBC1D1 (tre‐2/USP6, BUB2, cdc16 domain family member 1) (Taylor et al. 2008). AS160 and TBC1D1, a Rab‐GTPase‐activating proteins (Rab‐GAP), are currently recognized as the most distal signaling proteins that have been implicated in insulin‐stimulated glucose transport in skeletal muscle (Kramer et al. 2006; Peck et al. 2009; An et al. 2010; Chen et al. 2011).

A single bout of acute exercise causes GLUT4 translocation independently of the insulin signaling pathway to increase muscle glucose uptake (Ploug et al. 1984; Nesher et al. 1985; Lee et al. 1995; Yeh et al. 1995). This exercise effect on glucose uptake is evident during and immediately after exercise, but it reverses progressively, with little or no residual effects measured 2–3 h after the cessation of exercise in rats (Wallberg‐Henriksson et al. 1988; Cartee et al. 1989). As the exercise effect on insulin‐independent glucose uptake subsides, there is a substantial increase in the insulin action of the glucose uptake in skeletal muscles (Richter et al. 1982; Garetto et al. 1984; Wallberg‐Henriksson et al. 1988). This postexercise increase in the insulin action of the glucose uptake is attributable to greater insulin‐stimulated GLUT4 translocation to the cell surface (Hansen et al. 1998). However, there is a great deal of evidence that elevated insulin‐stimulated glucose uptake after exercise is not accompanied by enhanced insulin signaling at proximal steps ranging from the insulin receptor to Akt, suggesting that a more distal event is critical for increased postexercise insulin action (Bonen et al. 1985; Treadway et al. 1989; Hansen et al. 1998; Wojtaszewski et al. 2000; Arias et al. 2007; Koshinaka et al. 2008).

Skeletal muscles are composed of different muscle fiber types, that is, slow‐twitch type I and fast‐twitch type II muscle fibers, with different metabolic characteristics. Numerous previous studies found that prior exercise increases the insulin‐stimulated glucose uptake in both muscles composed of predominantly slow‐twitch type I fibers and muscles composed mainly of fast‐twitch type II fibers (Richter et al. 1984; Cartee et al. 1989; Hansen et al. 1998; Hamada et al. 2006; Tanaka et al. 2007). In addition, previous studies demonstrated that, in rat epitrochlearis muscle (which is composed of predominantly fast‐twitch type II fibers), the phosphorylation of both AS160 and TBC1D1 is increased immediately postexercise (Funai et al. 2009). Moreover, the phosphorylation of AS160 but not TBC1D1 remains elevated for up to 3 and 27 h after the cessation of exercise (Arias et al. 2007; Funai et al. 2009, 2010; Schweitzer et al. 2012). These results led to the idea that the persistent increase in AS160 phosphorylation but not in TBC1D1 phosphorylation after the cessation of exercise is important for the increased postexercise insulin action of glucose uptake in fast‐twitch muscle.

On the other hand, it is not clear whether increased phosphorylation of AS160 and/or TBC1D1 is involved with a postexercise increase in the insulin action of glucose uptake in slow‐twitch muscle. In this study, we evaluated the postexercise phosphorylation status of AS160 and TBC1D1 in soleus muscle consisting mainly of slow‐twitch type I muscle fibers.

## Material and Methods

### Materials

Antibodies against phospho‐Akt Ser^473^ (#9271), phospho‐Akt Thr^308^ (#9275), phospho‐TBC1D1 Thr^590^ (#6927), total Akt (#9272), and total acetyl CoA carboxylase (ACC, #3662) were purchased from Cell Signaling Technology (Beverly, MA). Antibodies against phospho‐AS160 Thr^642^ (#07‐802), phospho‐ACC Ser^79^ (#07‐303), phospho‐TBC1D1 Ser^237^ (#07‐2268), and total AS160 (#07‐741) were from Millipore (Temecula, CA). Anti‐phospho‐AS160 Ser^588^(#3028P2) was from Symansis Limited (Timaru, New Zealand). Anti‐GLUT4 antibody (#4670‐1704) was from Bio‐Rad AbD Serotec (Oxford, UK). HRP‐conjugated anti‐rabbit IgG was from Biosource International (Camarillo, CA). HRP‐conjugated anti‐sheep IgG was from Millipore. Enhanced chemiluminescence reagents (ECL, ECL Plus, and ECL Prime) were obtained from GE Healthcare Life Sciences (Uppsala, Sweden). All other reagents were obtained from Sigma (St. Louis, MO).

### Treatment of animals

This research was approved by the Animal Studies Committee of Niigata University of Health and Welfare. Three‐week‐old male Wistar rats were obtained from CLEA Japan (Tokyo). Animals were maintained in individual cages and fed a standard rodent chow diet and water ad libitum. All rats were accustomed to a rodent treadmill (Natsume Seisakusyo, Tokyo) for 10 min/day for 2 days before the experiment. We divided the rats (40–50 g) into two groups: a resting control group and an exercise group.

Rats in both resting control and exercise group were fasted from 12:00 pm of the experiment day. Rats in the exercise group ran on the treadmill for 180 min at 9 m/min on a 15% incline. The exercised rats finished running at 6:00 pm. Following the exercise protocol, the rats were killed by cervical dislocation either immediately or 2 h after the completion of the exercise. The resting control group was time‐matched with the exercised group, with the tissues of the control rats being collected at the same time as those of the exercised rats.

In the animals that were killed immediately after exercise, the soleus muscles consisting predominantly of slow‐twitch type I muscle fibers were rapidly dissected out. A portion of muscles were blotted, clamp‐frozen in liquid nitrogen for the subsequent western blot analysis as described below for the measurement of the insulin‐independent phosphorylation levels of signaling proteins. Other portion of muscles were incubated with shaking for 20 min at 30°C in 3 mL of oxygenated Krebs–Hensleit buffer (KHB) containing 40 mmol/L mannitol, 0.1% radioimmunoassay (RIA)‐grade bovine serum albumin (BSA) in the absence of insulin. Flasks were gassed continuously with 95% O_2_–5% CO_2_ during incubation. After incubation, these muscles were used for the measurement of insulin‐independent 2DG uptake.

The animals to be killed 2 h after the cessation of exercise were returned to their cages and remained fasting for another 2 h. These animals were then killed and the soleus muscles from both legs were dissected out. All muscles were incubated for 20 min at 30°C in 3 mL of oxygenated KHB containing 40 mmol/L mannitol, 0.1% RIA‐grade BSA. During this step, one muscle from each rat was incubated with 50 μU/mL of insulin, and the contralateral muscle was incubated without insulin. After incubation, muscles from a portion of animals were used for the measurement of basal and insulin‐stimulated 2DG uptake. Muscles from the other portion of animals were blotted, clamp‐frozen in liquid nitrogen for the subsequent western blot analysis as described below for the measurement of basal and insulin‐stimulated phosphorylation levels of signaling proteins.

### Measurement of 2DG uptake

We used 2DG to measure the rate of muscle glucose uptake based on a described method (Ueyama et al. 2000; Koshinaka et al. 2008, 2009). After a 20‐min incubation as described earlier, soleus muscles were incubated for 20 min at 30°C in 3 mL of KHB containing 8 mmol/L 2DG, 32 mmol/L mannitol, and 0.1% BSA in the presence or absence of purified human insulin (if present in the previous incubation). The flasks were gassed continuously with 95% O_2_–5% CO_2_ during the incubation. After the incubation, the muscles were blotted and then clamp‐frozen in liquid nitrogen. The concentrations of 2‐deoxyglucose‐6‐phosphate (2DG6P) in muscles was determined as described previously (Koshinaka et al. 2008, 2009).

### Western blot analysis

Soleus muscles were homogenized in ice‐cold buffer containing 50 mmol/L HEPES (pH 7.4), 150 mmol/L NaCl, 10% glycerol, 1% Triton X‐100, 1.5 mmol/L MgCl_2_, 1 mmol/L EDTA, 10 mmol/L Na_4_P_2_O_7_, 100 mmol/L NaF, 2 mmol/L Na_3_VO_4_, 2 mmol/L PMSF, aprotinin (10 μg/mL), leupeptin (10 μg/mL), and pepstatin (5 μg/mL) (Margolis et al. 1990). The homogenates were then rotated end‐over‐end at 4°C for 60 min, and centrifuged at 4000*g* for 30 min at 4°C. Aliquots of the supernatants were treated with 2× Laemmli sample buffer containing 100 mmol/L dithiothreitol. For the measurement of phospho‐AS160, total‐AS160, phospho‐TBC1D1, total‐TBC1D1, phospho‐ACC, and total‐ACC, the samples were subjected to 5% sodium dodecyl sulfate‐polyacrylamide gel electrophoresis (SDS‐PAGE). For the measurement of phospho‐Akt, total‐Akt, total‐Akt, and GLUT4, the samples were run on 10% SDS‐PAGE.

The resolved proteins were then transferred to PVDF membranes, and blocked in 5% nonfat dry milk in Tris‐buffered saline containing 0.1% Tween 10 (TBST), pH 7.5. After the blocking, the membranes were rinsed in TBST and then incubated overnight with the appropriate antibody at 4°C, followed by rinsing in TBST and incubation for 120 min with HRP‐conjugated anti‐rabbit IgG or HRP‐conjugated anti‐sheep IgG. Antibody‐bound protein was visualized by enhanced chemiluminescence (ECL, ECL Plus, or ECL Prime) with the intensity of the bands quantified by densitometry.

### Statistical analysis

Data are expressed as means ± SE. Differences were determined either using an unpaired Student's *t*‐test or a one‐way analysis of variance (ANOVA) with a subsequent Fisher's least significant difference method. Differences between groups were considered significant when *P* < 0.05.

## Results

### Insulin‐independent glucose uptake immediately postexercise

**Figure 1.**
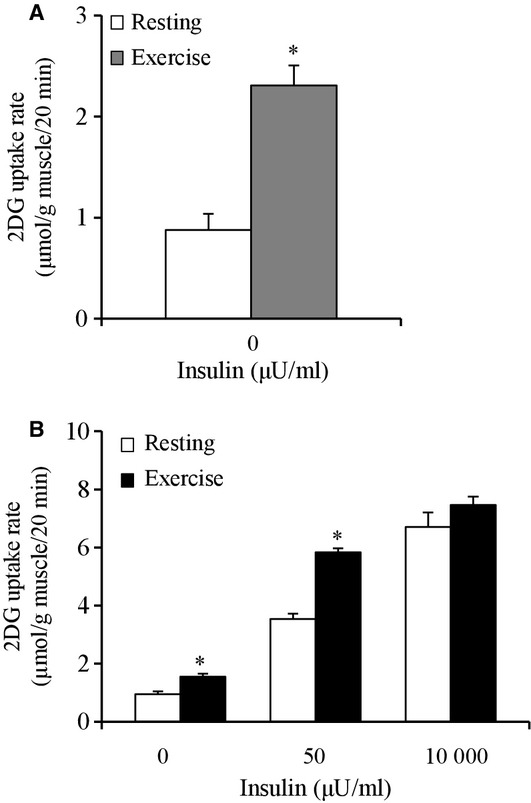
(A) Insulin‐independent glucose uptake in rat soleus muscles immediately after treadmill exercise (180 min at 9 m/min). Details are provided in the Materials and Methods section. Muscles were dissected out immediately after exercise or a time‐matched resting period. Values are mean ± SE (*n* = 7–8). *P < 0.05 versus resting. (B) Basal and insulin‐stimulated glucose uptake in rat soleus muscles 120 min after treadmill exercise (180 min at 9 m/min). Muscles were dissected out 120 min after exercise or a time‐matched resting period. All muscles were incubated in glucose‐free medium in the absence or presence (50 or 10,000 μU/mL) of insulin for 20 min, followed by measurement of 2DG uptake. Values are means ± SE (*n* = 8). *P < 0.05 versus resting with same insulin concentration.

Soleus muscles were dissected out from rats immediately after the cessation of 180 min of 9 m/min treadmill exercise and used for the measurement of the insulin‐independent 2DG uptake. The insulin‐independent 2DG uptake in the muscles of the exercised rats was 2.6‐fold higher compared to the values in the nonexercised rats (*P* < 0.05, Fig. 1A).

### Insulin‐stimulated glucose uptake and GLUT4 protein abundance at 2 h postexercise

Soleus muscles were dissected out from rats at 2 h after the cessation of 180 min of 9 m/min treadmill exercise and used for the measurement of basal and insulin‐stimulated 2DG uptake. Basal (0 μU/mL) 2DG uptake at 2 h postexercise was 63% higher compared to the values of the nonexercised rats (*P* < 0.05, Fig. 1B). The 2DG uptake in muscles stimulated by submaximal (50 μU/mL) insulin was 65% higher compared to the values of the nonexercised rats (*P* < 0.05, Fig. 1B). In contrast, 2DG uptake in muscles stimulated by the maximal (10,000 μU/mL) insulin was not significantly different between the exercised and nonexercised muscles at 2 h after 180 min of 9 m/min exercise (Fig. 1B).

When we calculated the submaximal (50 μU/mL) insulin‐stimulated increase above the basal level of 2DG uptake by subtracting the basal value from the insulin‐stimulated value, calculated ∆ insulin for the 2DG uptake in the exercised muscles was 65% greater compared to the values of the nonexercised muscles (*P* < 0.05, nonexercise: 2.59 ± 0.16 μmol/g muscle/20 min, *n* = 8; exercise: 4.28 ± 0.18 μmol/g muscle/20 min, *n* = 8).

As it has been well established that an abundance of GLUT4 protein is a determinant of insulin‐stimulated glucose uptake in skeletal muscles, we examined the effect of 180 min of 9 m/min exercise on the GLUT4 protein expression. There was no significant change in total GLUT4 abundance in the soleus muscles at 2 h postexercise (nonexercise: 100 ± 7 arbitrary units [AU], *n* = 8; exercise: 105 ± 4 AU, *n* = 8).

### Phosphorylation of Akt Ser473, Akt Thr308, AS160 Thr642, AS160 Ser588, TBC1D1 Thr590, TBC1D1 Ser237, and ACC Ser79 immediately after exercise

We dissected out the soleus muscles from rats immediately after the cessation of 180‐min exercise at 9 m/min and used them for measuring the insulin‐independent phosphorylation state of signaling molecules that are involved in GLUT4 translocation.

**Figure 2.**
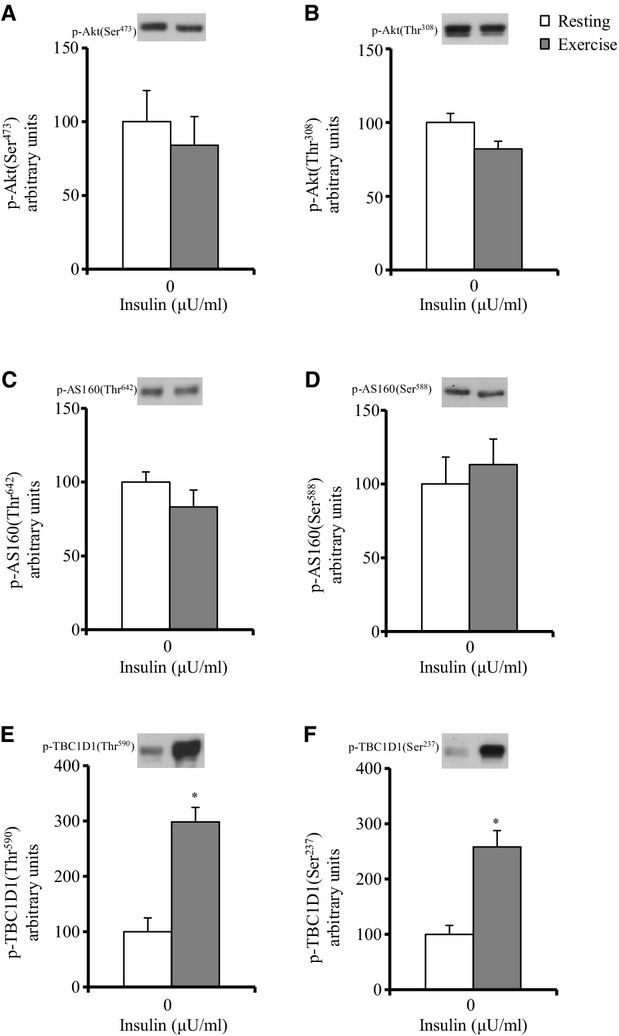
Phosphorylation of Akt, AS160, and TBC1D1 in rat soleus muscles immediately after treadmill exercise (180 min at 9 m/min). Details are provided in the text. Frozen muscles were used to measure the phosphorylation of Akt Ser473 (A), Akt Thr308 (B), AS160 Thr642 (C), AS160 Ser588 (D), TBC1D1 Thr590 (E), and TBC1D1 Ser237 (F). Values are mean ± SE (*n* = 7–8). *P < 0.05 versus resting.

The phosphorylation of Akt at both Ser473 and Thr508 is required for maximal enzyme activation (Alessi et al. 1996). Therefore, we estimated the Ser473 and Thr308 phosphorylation of Akt as indexes of the Akt activation level. Immediately postexercise, no significant effects of exercise on the insulin‐independent phosphorylation of Akt Ser473 or Akt Thr308 were observed (Fig. 2A and B). There was also no significant difference between the nonexercised and exercised groups for total Akt protein abundance (nonexercise: 100 ± 6 arbitrary units [AU], *n* = 8; exercise: 95 ± 5 AU, *n* = 8).

The phosphorylation of AS160 on both Thr642 and Ser588 is important for GLUT4 translocation in response to insulin's activation of Akt (Sano et al. 2003). Immediately postexercise, there were no significant effects of exercise on the insulin‐independent phosphorylation of AS160 Thr642 and AS160 Ser588 (Fig. 2C and D). There was also no significant difference between the exercise and nonexercise control groups for total AS160 protein abundance (nonexercise: 100 ± 5 AU, *n* = 8; exercise: 103 ± 3 AU, *n* = 8).

The phosphorylation of TBC1D1 on the Akt‐targeted phosphomotif Thr590 is probably involved in insulin‐stimulated GLUT4 translocation (Taylor et al. 2008; Peck et al. 2009; Vichaiwong et al. 2010). The phosphorylation of TBC1D1 on the AMPK‐targeted phosphomotif Ser237 is likely involved in insulin‐independent contraction‐stimulated GLUT4 translocation (Vichaiwong et al. 2010). We found that immediately postexercise, the insulin‐independent phosphorylation of TBC1D1 Thr590 and TBC1D1 Ser237 in the muscles of exercised rats were increased by threefold and 2.6‐fold, respectively, compared to the muscles of the nonexercised rats (*P* < 0.05, Fig. 2E and F).

The phosphorylation of ACC Ser79 reflects the AMPK activation level, since active AMPK phosphorylates ACC at the Ser79 site (Winder 2001; Hardie and Sakamoto 2006). We found that, immediately postexercise, the insulin‐independent phosphorylation of ACC Ser79 in the muscles of the exercised rats was increased by 2.8‐fold compared to the muscles of the nonexercised rats (*P* < 0.05, nonexercise: 100 ± 12 AU, *n* = 7; exercise: 277 ± 10 AU, *n* = 8). There was no significant difference between the exercise and nonexercise groups for total ACC protein abundance (nonexercise: 100 ± 8 AU, *n* = 8; exercise: 82 ± 7 AU, *n* = 8).

### Phosphorylation of Akt Ser473, Akt Thr308, AS160 Thr642, AS160 Ser588, TBC1D1 Thr590, and TBC1D1 Ser237 at 2 h postexercise

Soleus muscles were dissected out from rats 2 h after the cessation of 180‐min exercise (9 m/min) and used for the measurement of the insulin‐independent and ‐dependent phosphorylation state of signaling molecules that are involved in GLUT4 translocation.

**Figure 3.**
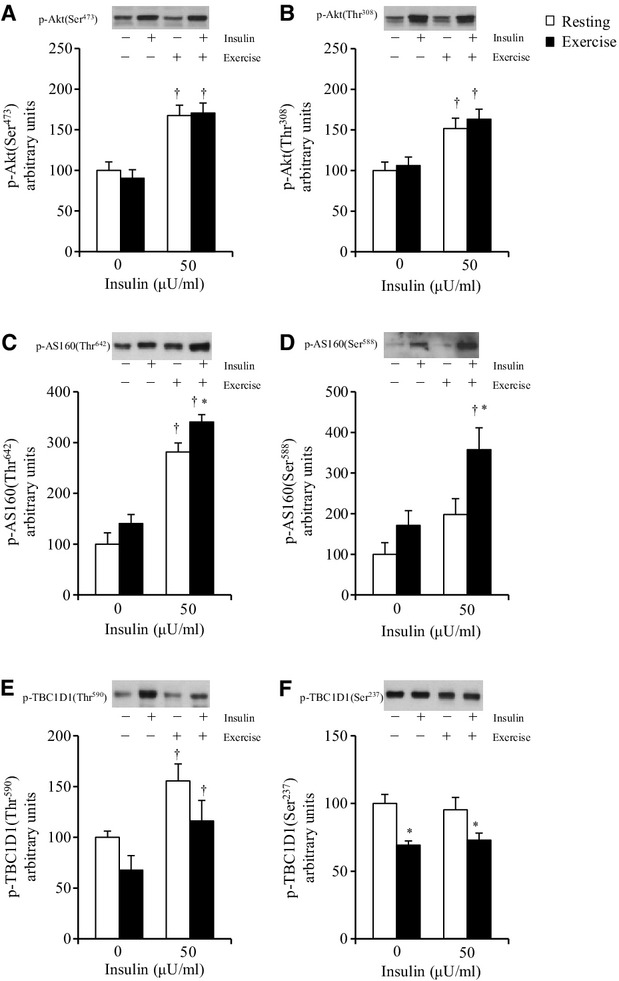
Phosphorylation of Akt, AS160, and TBC1D1 in rat soleus muscles 2 h after treadmill exercise (180 min at 9 m/min). See the Materials and Methods section for details. Frozen muscles were used to measure the phosphorylation of Akt Ser473 (A), Akt Thr308 (B), AS160 Thr642 (C), AS160 Ser588 (D), TBC1D1 Thr590 (E), and TBC1D1 Ser237 (F). Values are mean ± SE, (A–D) *n* = 7–8; (E) *n* = 6–7; (F) *n* = 7–8. ^†^*P* < 0.05 versus 0 *μ*U/mL insulin. *P < 0.05 versus resting with same insulin concentration.

When the muscles were dissected out at 2 h postexercise, no significant effect of exercise on the insulin‐independent phosphorylation of either Akt Ser473 or Thr308 was observed (Fig. 3A and B). At 2 h postexercise, the Akt Ser473 and Akt Thr308 phosphorylations were increased with submaximal (50 μU/mL) insulin treatment in the muscles of both nonexercised and exercised rats (Fig. 3A and B). There was no exercise effect on the phosphorylation of either Akt Ser473 or Thr308 in muscles treated with submaximal (50 μU/mL) insulin (Fig. 3A and B). We observed no significant difference between the 2 h postexercise and the nonexercised groups for total Akt protein abundance (resting: 100 ± 6 AU, *n* = 8; exercise: 92 ± 7 AU, *n* = 8).

When muscles were dissected out 2 h postexercise, there was no exercise effect on the insulin‐independent basal phosphorylation of either AS160 Thr642 or AS160 Ser588 (Fig. 3C and D). AS160 Thr642 phosphorylation was increased with submaximal (50 μU/mL) insulin treatment in the muscles of both nonexercised and exercised rats (Fig. 3C). On the other hand, the AS160 Ser588 phosphorylation was not significantly increased with submaximal (50 μU/mL) insulin treatment in the muscles of the nonexercised rats but it was significantly increased in those of the exercised rats (*P* < 0.05, Fig. 3D). In addition, at 2 h postexercise, phosphorylation of AS160 Thr642 and AS160 Ser588 in the exercised muscles treated with the submaximal (50 μU/mL) insulin was 21% and 69% greater, respectively, compared to the nonexercised muscles treated with insulin (*P* < 0.05, Fig. 3C and D). There was no significant difference between the exercised and nonexercised rats for total AS160 protein abundance at 2 h postexercise (nonexercise: 100 ± 4 AU, *n* = 8; exercise: 102 ± 6 AU, *n* = 8).

When we calculated the submaximal (50 μU/mL) insulin‐stimulated increase above the basal level of AS160 phosphorylation by subtracting the basal value from the insulin‐stimulated value, calculated ∆ insulin for phosphorylation of AS160 Thr642 in the exercised muscles was not significantly greater compared to the values of the nonexercised muscles (nonexercise: 182 ± 21 AU, *n* = 8; exercise: 200 ± 20 AU, *n* = 8). Calculated ∆ insulin for phosphorylation of AS160 Ser588 in the exercised muscles was also not significantly greater compared to the values of the nonexercised muscles (nonexercise: 98 ± 24 AU, *n* = 7; exercise: 186 ± 45 AU, *n* = 7).

When muscles were dissected out at 2 h postexercise, there was no significant exercise effect on the insulin‐independent basal phosphorylation of TBC1D1 Thr590 (Fig. 3E). The insulin‐independent basal phosphorylation of TBC1D1 Ser237 was 30% lower in the exercised muscles compared to the nonexercised muscle (*P* < 0.05, Fig. 3F). Thus, at 2 h postexercise, there was no residual increase in the insulin‐independent phosphorylation of either TBC1D1 Thr590 or TBC1D1 Ser237 in the muscles of the exercised rats compared to those of the nonexercised rats (Fig. 3E and F). Moreover, at 2 h postexercise, the TBC1D1 Thr590 phosphorylation was significantly increased with submaximal (50 μU/mL) insulin treatment in the muscles of both nonexercised and exercised rats (*P* < 0.05, Fig. 3E), whereas there was no effect of insulin treatment on TBC1D1 Ser237 phosphorylation (Fig. 3F). There was no significant exercise effect on the phosphorylation of TBC1D1 Thr590 in muscles stimulated by submaximal (50 μU/mL) insulin (Fig. 3E). Phosphorylation of TBC1D1 Ser237 in the exercised muscles treated with the submaximal (50 μU/mL) insulin was 23% lower compared to the nonexercised muscles treated with insulin (*P* < 0.05, Fig. 3F).

## Discussion

Our present findings revealed that in rat slow‐twitch soleus muscle, the submaximal (50 μU/mL) but not maximal (10000 μU/mL) insulin‐stimulated glucose uptake was enhanced at 2 h postexercise (Fig. 1B), demonstrating that insulin sensitivity is enhanced at 2 h postexercise in this muscle. Our results also showed that 2 h postexercise, when insulin sensitivity was enhanced, both the Thr642 and Ser588 phosphorylation of AS160 was elevated in soleus muscle, while TBC1D1 Thr590 phosphorylation was unaffected (Fig. 3C–E). These results support the idea that the increased phosphorylation of AS160, but not TBC1D1, can account for the enhanced postexercise insulin sensitivity in slow‐twitch muscle. We also found that neither the Akt Ser473 nor the Akt Thr308 phosphorylation of insulin‐stimulated soleus muscle was increased by prior exercise (Fig. 3A and B). Thus, the elevated AS160 phosphorylation was not accompanied by enhanced insulin signaling at proximal steps ranging from the insulin receptor to Akt. There are at least four kinases (Akt, AMPK, SGK, and RSK) known to phosphorylate AS160 (Geraghty et al. 2007). Moreover, the serine/threonine phosphatases (PP1, PP2A, PP2B, and PP2C) were found to be able to dephosphorylate AS160 at both the Thr642 and Ser588 phosphosites (Schweitzer et al. 2012). It may be possible that the increased activity of some kinase(s) other than Akt or the decreased activity of some phosphatase(s) is involved in the postexercise increase in AS160 phosphorylation and the insulin action of the glucose uptake in soleus muscle.

When we calculated the insulin‐stimulated increase above the basal level of AS160 phosphorylation by subtracting the basal values from the insulin‐stimulated values, calculated ∆ insulin for the phosphorylation of AS160 (Thr642 and Ser588) was not significantly elevated in slow‐twitch soleus muscle after exercise (see Results). Therefore, increased postexercise AS160 phosphorylation in the presence of insulin may be attributable to the greater baseline values (without insulin) rather than an insulin‐stimulated increase above the basal levels of AS160 phosphorylation, although we did not observe a statistical significant elevation of basal AS160 phosphorylation (Fig. 3C and D). Previous research from Cartee et al.'s group on rat epitrochlearis muscle (which is composed of predominantly fast‐twitch type II fibers) also demonstrated that increased postexercise AS160 phosphorylation in the presence of insulin was entirely attributable to the greater baseline values (without insulin) rather than ∆ insulin (Arias et al. 2007; Funai et al. 2009, 2010; Schweitzer et al. 2012). What is a possible mechanism that could link the increased basal AS160 phosphorylation in the absence of insulin with increased insulin‐stimulated glucose uptake after exercise? In the basal state, AS160's active Rab GTPase‐activating protein domain is hypothesized to restrain the exocytosis of intracellular GLUT4 storage vesicles (Sano et al. 2003; Sakamoto and Holman 2008). The insulin‐stimulated phosphorylation of AS160 appears to relieve this restraint and allow GLUT4 to be recruited to the cell surface membranes. In this context, increased basal AS160 phosphorylation may attenuate AS160's inhibitory effect on GLUT4 translocation and render GLUT4 more sensitive to a subsequent insulin‐triggered translocation. Therefore, an increment in basal AS160 phosphorylation may be important for the increase in the insulin‐stimulated glucose uptake in exercised muscle.

In 2013, Xiao et al. (2013) published a study demonstrating that a postexercise increase in the insulin action of glucose uptake was not accompanied by increased AS160 phosphorylation in slow‐twitch soleus muscle of old rats (24‐month‐old). Their results are not consistent with our present evidence that prior exercise increases the AS160 phosphorylation in insulin‐stimulated soleus muscle. We do not presently know the reason for this discrepancy. However, since we used soleus muscles from young rats (~1‐month‐old) in our present study, it seems reasonable to suspect that the difference in the animals’ ages could be involved with the discrepancy between Xiao et al.'s findings and our present results. As animals usually have been restricted in small cages without exercise and provided with unlimited food, they become obese with aging. Therefore, old animals exhibit muscle insulin resistance for glucose uptake. Exercise might increase phosphorylation level of AS160 in insulin‐sensitive muscles of young animals but not in insulin‐resistant muscles of old animals. In addition, rats were exercised by swimming (90 min) in Xiao et al.'s study, whereas we exercised rats on a treadmill (180 min) in our present study. It might be possible that the discrepancy between both studies is due to difference in exercise protocol.

Previous studies have provided evidence of the enhanced insulin action of glucose uptake in rat skeletal muscle after the cessation of an acute bout of moderate (18 m/min)‐ or high (36 m/min)‐intensity prolonged treadmill exercise (Richter et al. 1982; Garetto et al. 1984; Zorzano et al. 1986). Since it was reported that the treadmill velocity corresponding to the lactate threshold (LT) in rats was between 17.5 and 20 m/min (Soya et al. 2007), the treadmill velocities of 18 and 36 m/min used in the previous studies are thought to correspond to the LT and above the LT, respectively. Thus, exercise at the LT or above the LT is considered to be effective for increasing the insulin action of muscle glucose uptake. However, in the present study the rats were exercised for 180 min on a treadmill at 9 m/min, which we consider below the LT (i.e., low‐intensity). To our knowledge, the present study is the first to demonstrate that low‐intensity exercise is effective for increasing the insulin action of the glucose uptake in rat slow‐twitch soleus muscle, as are moderate and high‐intensity exercise. We also found that less volume of low‐intensity exercise (90 min on a treadmill at 9 m/min) increased the insulin action of glucose uptake in rat soleus muscle (data not shown). These results provide a possible reason why low‐intensity physical activities such as mild walking, which can be easily and safely performed even by older people, is effective for the prevention and treatment of diabetes.

Our findings showed that in rat slow‐twitch soleus muscle, the glucose uptake in the absence of insulin was increased immediately after the cessation of low‐intensity exercise (Fig. 1A). This is likely due to the stimulating effect of muscle contractile activity on insulin‐independent GLUT4 translocation (Ploug et al. 1984; Nesher et al. 1985; Lee et al. 1995; Yeh et al. 1995). It is well established that the contraction effect may involve multiple molecules including the AMP‐activated protein kinase (AMPK) (Hayashi et al. 1998; Winder 2001). Moreover, previous studies demonstrated that muscle contraction causes the site‐specific phosphorylation of TBC1D1 via AMPK activation (Funai and Cartee 2009; Pehmøller et al. 2009; Frøsig et al. 2010; Vichaiwong et al. 2010), and TBC1D1 phosphorylation on AMPK sites regulates contraction‐stimulated glucose uptake (Vichaiwong et al. 2010). In the present study, the ACC phosphorylation on the Ser79 site (a commonly used indicator of AMPK activity) was increased immediately after the cessation of exercise (see Results). In addition, the TBC1D1 phosphorylation on the predicted AMPK phosphorylation site (Ser237) was also increased immediately postexercise (Fig. 2F). Thus, increased AMPK activation and the subsequent elevation of TBC1D1 Ser237 phosphorylation may explain the increased insulin‐independent glucose uptake immediately after low‐intensity exercise.

In conclusion, the results of our study demonstrated that, in rat slow‐twitch soleus muscle, AS160 phosphorylation in the presence of insulin was increased together with the enhanced insulin action of the glucose uptake at 2 h postexercise. The phosphosite of TBC1D1 (Thr590), which is possibly involved with insulin‐stimulated glucose uptake, did not increase phosphorylation at 2 h postexercise. We therefore suggest that the increased phosphorylation of AS160, but not TBC1D1, can account for the postexercise enhancement in the insulin action of the glucose uptake in rat slow‐twitch soleus muscle.

## Conflict of Interest

None declared.
